# Molecular determinants of neuroprotection in blood-brain interfaces of the cynomolgus monkey

**DOI:** 10.3389/fphar.2025.1523819

**Published:** 2025-03-12

**Authors:** Nathalie Strazielle, Sandrine Blondel, Joachim Confais, Rita El Khoury, Hugues Contamin, Jean-François Ghersi-Egea

**Affiliations:** ^1^ BIP Facility, Fluid Team, Lyon Neurosciences Research Center, INSERM U1028, UMR CNRS, University Lyon 1, Bron, France; ^2^ Brain-i, Lyon, France; ^3^ Cynbiose, Sainte-Consorce, France

**Keywords:** blood-brain barrier, blood-cerebrospinal fluid barrier, choroid plexus, efflux pumps, non-human primate, translational pharmacokinetics, transporters

## Abstract

The blood-brain barrier (BBB) formed by the cerebral microvessel endothelium and the blood-CSF barrier (BCSFB) formed by the choroid plexus epithelium impact the cerebral bioavailability of drugs and endogenous molecules that contribute to neuroinflammatory and neurodegenerative diseases. Species specificities in tight junction proteins and efflux transporters governing the barrier functions of these interfaces hamper the direct translation of pharmacokinetic and pathophysiological data from rodents to human. We defined the molecular composition of tight junctions and identified the efflux transporters present at the BBB and BCSFB of cynomolgus monkey to assess whether this species is a relevant alternative to rodents. Choroid plexuses, cerebral microvessels, cortex and cerebellum were isolated from adult cynomolgus monkeys, and analysed by RT-qPCR and immunohistochemistry. Results were compared with data available in the literature for rat and human. In monkeys as in rat and human, claudin-5 in the BBB and claudin-1, -2, -3 in the BCSFB were landmark tight junction proteins. ABCB1 was strictly associated with the BBB, and ABCC1 was predominant at the BCSFB compared to the BBB. The monkey, like human, differed from rat by the localization of ABCG2 protein in choroidal vessels, a low expression of ABCC4 and SLC22A8 in the BBB, and the presence of SLC47A1 at the BCSFB. While the main characteristics of brain barriers are common to all three species, cynomolgus monkey and human share specificities in the expression and localization of selected claudins and efflux transporters that are not met in rat.

## Introduction

The blood-brain interfaces protect the brain from blood-borne deleterious molecules, and coupled with cerebrospinal fluid (CSF) circulation, they maintain the homeostasis necessary for normal brain function. They include the blood-brain barrier proper (BBB), located at the endothelium of the cerebral microvessels and larger vessels, and the blood-CSF barrier (BCSFB) formed by the epithelium of the choroid plexuses which are located within the brain ventricles. The arachnoid membrane located downstream of the CSF flow also forms a barrier between the outer CSF and the blood. To some extent, the pericytes and astrocytes appended to the cerebral microvessels participate to the establishment of the barrier properties associated with the cerebral endothelium. The barrier phenotype of these interfaces results primarily from the presence of continuous tight junctions that seal the barrier cells together and prevent the non-specific paracellular diffusion of blood-borne molecules. Specific claudins, occludin and adaptor tight junction proteins make up these tight junctions. Multispecific efflux transporters that restrict blood-to-brain/CSF, or favor brain/CSF-to-blood, fluxes across barrier cells also contribute to this phenotype. Three families of ATP-binding Cassette (ABC) transporters, ABCB, ABCC and ABCG, and selected solute carriers (SLC), mainly of the SLC22, SLCO, and SLC47 families are especially involved in these efflux processes (Strazielle and Ghersi-Egea, 2013).

These different barriers mechanisms prevent blood-borne toxic molecules from entering the central nervous system (CNS), and participate in the elimination of potentially deleterious cerebral endogenous compounds, thus contributing to cerebral homeostasis. Unsurprisingly, dysfunctions of brain barriers are involved in the pathophysiology of various degenerative and inflammatory CNS diseases (Hubert et al., 2019; Sweeney et al., 2019). The barrier mechanisms also strongly impact the cerebral pharmacokinetic of numerous drugs and impede their cerebral delivery (Sugiyama et al., 1999; Goncalves et al., 2018).

Differences exist between human and the species used in pharmacotoxicological studies with respect to blood-brain interfaces attributes. These differences bear on the protein composition of tight junctions, as illustrated by claudin-5 whose immunoreactivity was reported in epithelial cell tight junctions of the choroid plexus in human, but not in rat (Mollgard et al., 2017; Virag et al., 2017). They also bear on the level of expression or substrate specificity of many efflux transporters at blood-brain interfaces. Among those differences, the level of ABCG2 protein relative to that of ABCB1 at the BBB is higher in human as compared to rodent, hence the impact of ABCG2 on brain drug delivery relative to that of ABCB1 is likely to be more important in human than in rodent (Shawahna et al., 2011). ABCG2 was reported absent in human and rat choroidal epithelium, but was detected in adult mouse epithelium (reviewed in Strazielle and Ghersi-Egea, 2015). The expression at the BBB of ABCC2, thought to be implicated in the mechanisms underlying resistance to antiepileptic drugs in epileptic patients (Kubota et al., 2006), may also display species specificities (discussed in Kubota et al., 2006; Dauchy et al., 2008). Organic anion-transporting polypeptides (OATPs) encoded by *SLCO* genes display a high degree of species variability that prevents the straightforward superposition of rodent and human OATP sub-family classification (Hagenbuch and Meier, 2004), and therefore the extrapolation of OATP-dependent transport data acquired in rodent to human.

This variability in the molecular effectors determining barrier functions needs to be considered when animal studies, especially those performed in rodents, are used to predict blood-brain transport processes and cerebral drug pharmacokinetic parameters for human. Non-human primates (NHP), in particular the cynomolgus monkey, have been considered to be more predictive species for neuropharmacological, toxicological and pathophysiological studies than rodents (Bontrop, 2001). Their genetic homology with humans is high, with 97% of the DNA sequences being identical between the two species. In addition, the CSF turnover rates of macaques and humans are very similar, and differ markedly from that of rodents, strengthening the relevance of the NHP model in cerebral pharmacokinetic studies (Barten et al., 2017). Several breeding colonies have been developed, providing access to this relevant model for pharmacokinetic studies. However, the distinctive features of blood-brain interfaces in cynomolgus monkeys have so far not been thoroughly investigated.

The present work provides a comprehensive analysis of the features that differentiate the two main blood-CNS interfaces with respect to their barrier functions in cynomolgus monkeys. We used a combination of immunohistochemistry on brain tissues and targeted RT-qPCR analyses of isolated cerebral microvessels and choroid plexuses to investigate the molecular attributes specifically relevant to the bioavailability of cerebral drugs and environmental compounds, and to neuroprotection, i.e., proteins of the tight junctions, ABC multispecific efflux transporters, and multidrug and toxin extrusion proteins encoded by *SLC47*, *SLC22* and *SLCO* genes. We favored this approach rather than performing an RNA sequencing analysis in view of the large interindividual variation expected from non-human primates compared to laboratory rodent species. Our study permitted to analyze multiple animals at an acceptable cost and to generate information on the differential localization of barrier attributes between the BBB and the BCSFB. Reports comparing these two interfaces for their neuroprotective attributes in other species are scarce. We previously conducted a similar study centered on tight junction proteins in rat (Kratzer et al., 2012). Other than that, most studies focus only on isolated capillaries or on isolated choroid plexuses. We nonetheless provide a tentative comparison of our data in monkey to those available in the literature for rodent and human.

## Methods

### Animals

2 juvenile and nine adult cynomolgus monkeys (*Macaca fascicularis)*, imported from Le Tamarinier (Mauritius), or Nafovanny (Vietnam) comprising four males and seven females were included in the study. The animals were part of other studies (Table 1) and were socially housed in primate cages under controlled conditions of humidity, temperature, and light (12-h light/12-h dark cycle, light on at 8.00 a.m.), within a dedicated primate facility. Primate diet was provided daily in amounts appropriate for the size and age of the animals. Fresh fruits were also given to the animals daily, and unsweetened treats were scattered in the litter as part of the Testing Facility Environmental Enrichment Program. Tap water was available *ad libitum* to each animal. Animal care was supervised by veterinarians experienced in NHP husbandry. Following acceptance of the study design by the IACUC for NHP experiments (National Veterinary School of Lyon, Lyon, France), brains were collected from animals undergoing experiments carried out in accordance to the European Communities Council Directive (2010/63/EU) for the care of laboratory animals. The eleven cynomolgus monkeys and their inclusion in the different aspects of the study are described in Table 1.

**TABLE 1:** Description of cynomolgus monkeys included in the study.

Case	Age (years)	Sex	Origin	Clinical history	Pharmacological treatment	Sample use
1	5.0	M	Mauritius	Dental surgery	Bone-derived neurotrophic factor, hyaluronic acid (local injection)	a, c
2	3.4	F	Mauritius	Knee arthrosis	Synovial injection of cartilage-repairing agent	a, c
3	3.3	F	Mauritius	Knee arthrosis	Synovial injection of cartilage-repairing agent	a, c
4	4.7	F	Mauritius	Knee arthrosis	Synovial injection of cartilage-repairing agent	a, c
5	4.7	F	Mauritius	Knee arthrosis	Synovial injection of cartilage-repairing agent	a
6	4.6	F	Mauritius	Knee arthrosis	Synovial injection of cartilage-repairing agent	a
7	4.6	F	Mauritius	Knee arthrosis	Synovial injection of cartilage-repairing agent	a, b, c, d
8	9.0	M	Mauritius	Catheter implantation in portal vein, vena cava	Local (antibiotics), and limited dosing with benzodiazepine	a, b
9	5.5	F	Mauritius		Antiaggregant antibodies (IV, 1 year before euthanasia), aspirin, small molecule against Parkinson Disease, cholesterol (IV)	d
10	4.5	M	Vietnam	Exposure to immunogenic antigens (IM) Bone marrow sampling	Vaccine candidate (IM), non-absorbable antibiotic drug (oral, 1 year before euthanasia)	d
11	4.1	M	Vietnam		Vaccine candidate (IM), non-absorbable antibiotic drug (oral, 1 year before euthanasia)	d

a: qRT-PCR (microdissected choroid plexuses, cerebral cortices and cebebellum); b: qRT-PCR, and γGT, enzymatic activity (isolated microvessels), c: immunohistochemistry. d: glutathione-S-transferase enzymatic measurements (cerebral cortices and choroid plexuses). IV: intravenous; IM: intramuscular.

### Tissue sampling

Animals were euthanized under deep anaesthesia (intramuscular injection of Ketamine and Midazolam) with 4 g of intravenous pentobarbital (Dolethal, Vetoquinol). Brains were collected and quickly immerged in cold HBSS (Gibco^®^, Fisher Scientific, France). Lateral ventricle choroid plexuses (LVCP) and fourth ventricle choroid plexuses (4VCP), as well as pieces of meninge-free cortex and cerebellum were collected. Samples collected for gene expression analyses were snap-frozen in liquid nitrogen and kept at −80°C. For immunohistochemistry, additional tissue fragments were snap-frozen in isopentane at −50°C, embedded in Tissue-Tek (Sakura Finetek Europe, Netherland) and stored at −80°C. Other fragments were fixed in RCL2 fixative (Alphelys, France) before paraffin embedding. In two animals, freshly collected cortical gray matter was used for immediate microvessel isolation, or frozen at −80°C for further microvessel isolation.

### Microvessel isolation

Brain microvessels were isolated as described previously in details for rats (Gazzin et al., 2008). Briefly, an average of 5 g of cortical tissue was used for each preparation. The tissue was carefully cleaned from meninges and superficial blood vessels prior to microvessel isolation. Minced cortical tissue was homogenized in Krebs-Ringer buffer (KR) by mechanical homogenization, and an aliquot of homogenate was sampled for enzymatic measurement. The preparation was further diluted with KR supplemented with 1% (w/v) bovine serum albumin (BSA) (5/1, v/w of tissue), homogenized, and subjected sequentially to several steps of centrifugation, to a 70 kDa dextran gradient, and to filtration on sieves of decreasing mesh sizes. Microvessels were collected on a 40 µm-mesh sieve in 0.1% BSA-supplemented KR.

### Total RNA isolation

Total RNA was prepared from LVCP, 4VCP, cerebral cortex, cerebellum, and brain microvessel fractions using Qiagen RNeasy Micro and Mini kits (Qiagen, Valencia, CA, United States) according to the manufacter’s instructions. Tissue was homogenized in RLT buffer using Soft tissue homogenizing tubes in the Minilys tissue grinder (both from Bertin Technologies, France) prior to the column purification step. Digestion with proteinase K (Qiagen) was performed on choroidal and microvascular samples. All tissues were treated with DNAse as recommended in the kit instructions. RNA was quantified by OD measurement at 260 nm using a NanoDrop spectrophotometer (ThermoScientific, Baltimore, MA, United States) and quality was assessed with the Agilent 2100 Bioanalyzer (Agilent Technologies, Palo Alto, CA, United States). Only samples with an RNA integrity number above 7.8 were used in the study.

BioChain^®^ total RNA from cynomolgus monkey peripheral tissues (colon, liver, lung, and kidney) were purchased from Clinisciences (Nanterre, France) to be used as positive control tissues to define PCR conditions. All RNAs were reverse-transcribed using the iScript cDNA Synthesis kit (Bio-Rad, Marnes-la-Coquette, France).

### Quantitative PCR

Quantitative polymerase chain reaction (qPCR) was performed using the LightCycler FastStart-DNA Master SYBR Green I kit and the LightCycler^®^ 2.0.5 Instrument (Roche Diagnostics GmbH, Mannheim, Germany), or the LightCycler^®^ 480 SYBR Green I Master kit and the LightCycler^®^ 480 Instrument II (Roche). Primers were designed using NCBI Primer-BLAST or chosen from the NCBI probe bank. They were selected to generate amplicons with a length of 85–200 bp (Table 2). After an initial step of DNA polymerase activation at 95°C for 8 min, the following amplification conditions were used: 45 cycles of denaturation at 95°C for 10 s, annealing for 10 s and extension at 72°C for 10 s. A touchdown protocol was applied by setting the initial annealing temperature at 68°C and decreasing this value by 0.5°C for the next 12 cycles, and running all the remaining annealing steps at 62°. Melting-curve analysis was then performed to verify the amplification of a single product with a specific melting temperature. For genes analyzed using the LightCycler FastStart-DNA Master SYBR Green I kit, MgCl_2_ concentration was optimized. A negative PCR control without cDNA was included in all runs. A standard curve was generated for each gene of interest by nonlinear regression analysis of crossing points (threshold cycles, Ct) generated for at least five serial dilutions of a cDNA pool, using the LightCycler^®^ Software 4.1 and LightCycler^®^ 480 Software 1.5.1.

**TABLE 2:** List of primers used for qPCR and corresponding product lengths.

Gene name	Forward primer 5‘→ 3‘	Reverse primer 5‘→ 3‘	Size (bp)
*ABCB1*	CGG​TTT​GGA​GCC​TAC​TTG​GT	ATG​AAC​TGA​CTT​GCC​CCA​CG	109
*ABCC1*	TGG​ACT​TCG​TTC​TCA​GGC​AC	GGC​AGA​CTC​GTT​GAT​CCG​AA	125
*ABCC4*	GCC​CTC​ACT​GAA​ACA​GCA​AAA	TTA​AGG​TCG​AGG​GCT​GTC​CA	109
*ABCG2*	GAG​CCT​TCC​AAG​CGG​GAT​AA	CAC​CCC​CGG​AAA​GTT​GAT​GT	109
*CD31*	AGT​CAG​AGT​CGT​TCT​TGC​CG	GGC​CTT​GGC​TTT​CCT​CAG​AA	118
*CLDN1*	GCT​TCT​CTC​TGC​CTT​CTG​GG	TTT​TGG​ATA​GGG​CCG​TGG​TG	90
*CLDN 2*	AGC​ATG​CAG​GTT​GAA​TTG​CC	GGA​TCC​TCT​GAG​TCC​TGG​CT	134
*CLDN 3*	AGT​ACA​TGC​CCA​CCA​AGG​TC	AGA​CAT​AGT​CCT​TGC​GGT​CG	93
*CLDN 4*	TGT​GCC​TTG​CTC​ACC​GAA​A	CAA​ACC​CGT​CCA​TCC​ACT​CTG	105
*CLDN 5*	CTGGTGCTGTGCCTGGTG	CCCCTTCCAGGTGGTCTG	117
*CLDN 7*	TGT​ACA​AGG​GGC​TGT​GGA​TG	GGA​GAC​CAC​CAT​TAG​GGC​TC	125
*CLDN 8*	TTG​TTG​GAG​GAG​CCC​TGT​TC	TTG​TGC​GAT​GGG​AGG​GTA​TC	87
*CLDN 9*	AGG​GGC​ACA​TTT​TTG​TGG​GT	GAA​GCT​CAA​ATC​CTG​ACC​CCT	130
*CLDN 11*	TCA​TTC​TGC​TGG​CTC​TCT​GC	GGA​GTA​GCC​AAA​GCT​CAC​GA	92
*CLDN 12*	TTT​TGA​GCC​CTC​ATC​AAG​CT	CTC​TCC​CAT​GGC​TGG​ATA​AA	151
*CLDN 14*	ACA​GAG​GGA​GGA​ATA​AGA​GGA​GG	GCC​AAA​CTC​CCA​GGC​TAC​TTT	140
*CLDN 15*	AGA​AAG​ATG​GAC​TCG​TGG​GC	CCA​CGC​CTC​CTT​CAG​GAT​TT	115
*CLDN 16*	AGG​CAC​CCC​AGG​AAT​CAT​TG	AGC​CAA​CAG​GAC​CAA​CCA​AA	123
*CLDN 17*	TCT​GTA​CTT​CAA​GCA​AAC​AGA​AGC	TCC​CTT​CAA​TGC​CCC​AAC​TG	128
*CLDN 18*	TTT​GGT​GCA​GCT​CTG​TTC​GT	GGC​CCG​AGG​CAT​GAT​AAG​AA	136
*CLDN 19*	TGT​CAG​AGT​TAG​AAG​GGC​TTT​TGG	TTG​GTT​CGG​GGA​GAT​GTA​GGA	147
*CLDN 20*	CGA​CAG​CCA​GCA​TCG​TTA​AG	AGA​GAC​CCC​AGA​TAA​GGC​CA	117
*CLDN 22*	CTG​AAT​TTT​TTT​CCA​CCC​AC	AAT​CAG​GTT​AAA​TTC​TGA​ACA​TGT​T	124
*CLDN 23*	TTC​GTG​GGA​CCA​AAC​AGG​AC	AAG​CCC​GTC​ACT​CCC​TAA​GA	119
*DPAGT1*	TGT​CTT​TGC​AGC​CTC​ACA​GG	GCC​CTG​GCC​CAA​GTT​CTA​TC	122
*OCLN*	TGC​AAT​GAA​GTC​TCT​GAA​GTG​AAA​C	TCT​AAA​ATA​TGA​AAG​GCC​AGG​GAG​T	109
*SLC22A8*	GGG​CGT​AAG​TAA​CCT​GTG​GA	TTC​CAG​GTC​TTC​GAT​AGT​CT	187
*SLC47A1*	CTC​CTG​CCC​CAG​ATC​GTA​AC	GCA​GAG​CCT​ATC​ACC​CCA​AG	101
*SLC47A2*	GTC​AGG​ATC​CTA​GCC​ACC​AG	TCA​GAC​CCC​TCT​GAG​TGT​CA	197
*SLCO1A2*	TGT​CAG​CTT​GTC​TTG​CTG​GT	GGA​ACA​GTC​AGG​CCC​TTT​GT	137
*SLCO2A1*	TGTGCCCGCTCGGTCT	AGC​CCA​AAG​CGC​TTC​TCA​AT	128
*SLCO3A1*	TGG​CAT​CAC​CTA​CCT​GTC​TG	AAC​CAC​GGT​CGC​ATT​CTC​A	103
*SLCO4A1*	CTC​CAT​CTG​GCT​CCT​CCT​GAA	CTT​GGG​GCT​AAA​CGT​GGA​CAT	103
*SLCO2B1*	GTT​CAT​CGG​CCT​CCA​GTT​CT	TGG​TCC​TTG​CCT​CTT​TGT​CC	98
*TTR*	ATG​GGC​TCA​CAA​CTG​AGG​AG	CGT​TGG​CTG​TGA​ATA​CCA​CC	129
*Z O -1*	GAC​AGC​AGA​CCA​CGT​TAC​GA	GAA​GGG​TAG​GGC​TGG​GTT​TC	119
*Z O -2*	TGG​TTC​GGC​AGC​TTA​AAG​GA	CAT​GCG​GTC​TTC​AGG​GTC​AT	105

Ct values of unknown samples were then used to determine in each sample a relative cDNA concentration of the target gene. Possible sample-to-sample variations in reverse transcription efficiency and in qPCR processing were corrected by normalizing the data to the expression of the gene dolichyl-phosphate N-acetylglucosaminephosphotransferase 1 (*DPAGT1*).

In order to provide an index of abundance for the different tight junction proteins and transporter gene products, expression levels of all genes were estimated first in a reference sample, arbitrarily chosen as LVCP cynomolgus #2 as follows:

DPAGT1 efficiency DPAGT1Ct/ Target efficiency Target Ct

where efficiencies of amplification were calculated from the linear part of the standard curves using the LightCycler Software 4.1. The obtained gene values were normalized to *CLDN1* value set as 100 after correction for the differences in the size of the amplification products. Then for each target gene, the expression level for each sample was expressed relative to the value of the reference sample.

### Histology and immunohistochemistry

Hematoxylin (Biolyon 0942)–phloxin (Ral diagnostics 361470)–safran (BDH Gurr 35093) was used to stain nuclei, cytoplasm and connective tissue, respectively, according to the following sequence. Parafin sections were deparafinized in methylcyclohexane (three times for 5 min), rehydrated through a graded series of ethanol/water solutions (100%–0% of ethanol), and incubated in hematoxylin (0.5% w/v) for 5 min. After a 10-min wash in running water, sections were immersed for 15 s in hydrochloric alcool (3 drops of pure hydrochloric acid in 100 mL of absolute ethanol). After a 10-min wash in running water, sections were immersed for 3 s in phloxin (1% w/v). The slides were then immersed for 10 s in an alcoholic safran solution before being rinsed in 100% ethanol. Sections were rinsed quickly with water before being dehydrated through a graded series of ethanol/water solutions (70%–100% ethanol) followed by 3 baths of methylcyclohexane. Sections were examined with an Axioplan microscope (Zeiss, France).

For immunofluorescence labelling, frozen sections were fixed differently depending on the primary antibodies (Table 3). Fixation in 4% (w/v) paraformaldehyde in phosphate buffer was performed at room temperature for 10 min. Fixation in 1% (w/v) paraformaldehyde in phosphate buffer was performed at room temperature for 30 s. Fixation in methanol/acetone (50/50, v/v) was performed at −20°C for 5 min. Fixation in acetone was performed at −20°C for 8 min. Fixed sections were blocked for 1 h at room temperature in phosphate buffer saline (PBS, Euromedex, France) containing 0.2% BSA w/v, 10% normal goat serum v/v, 0.3% Triton X100 v/v for claudins (CLDN-1, -2, -3, -4, and -5). For all other primary antibodies, sections were blocked in a solution containing 5% BSA, 5% normal goat serum, 0.3% Triton X-100 in PBS. Sections were incubated overnight at 4°C in the blocking solution containing the primary antibody. The list of primary antibodies, with company names, product references and final concentrations is given in Table 3. Following three washes in the blocking solution, the sections were incubated with a secondary anti-rabbit, anti-rat or anti-mouse Alexa-conjugated antibody (Invitrogen, 2 μg/mL in blocking solution) for 1 h at room temperature. Nuclei were stained with 4′,6-Diamidine-2′-phenylindole dihydrochloride (Roche, 5 μg/mL in PBS) for 10 min at room temperature and sections were mounted with fluorescence mounting medium (F/TA-030-FM, Thermo Scientific). Negative controls were performed by omitting the primary antibody. Fluorescent immunolabelling was observed using an Axio Imager M2 fluorescence microscope (Zeiss).

**TABLE 3:** Antibodies used for immunohistochemical studies.

Protein name	Company	Reference/RRID	Concentration (µg/mL)	Fixing solution used
CLDN-1	Invitrogen	51-9000/ AB_2533916	0.625	Acetone/methanol (50/50)
CLDN-2	Invitrogen	51-6100/ AB_2533911	0.625	Acetone/methanol (50/50)
CLDN-3	Invitrogen	34-1700/ AB_2533158	0.625	Aceton/methanol (50/50)
CLDN-4	Gentaur	18-272-196247	0.666	4% paraformaldehyde in PBS
CLDN-5	Invitrogen	35-2,500/ AB_2533200	2	Acetone/methanol (50/50)
CLDN-5	Invitrogen	34-1600/ AB_2533157	0.5	Acetone/methanol (50/50)
MRP1 (A23)	Alexis	ALX-210-841/ AB_2076039	1	4% paraformaldehyde in PBS or acetone/methanol (50/50)
MRP4 (M4I-10)	Solvo	SB M4I 10 MAB/	7.5	100% acetone
PGP (C219)	Calbiochem	517310/ AB_564389	1.45	1% paraformaldehyde in PBS
ABCG2 (BXP-21)	Alexis	ALX-801-029/ AB_2220324	2.5	100% acetone

### Enzymatic activities

Gamma-glutamyltransferase activity was determined in tissue homogenates and microvessel preparations using a dual-beam Cary 100 spectrophotometer (Varian) with L-γ-glutamyl-3-carboxy-4-nitroanilide as a substrate, in the presence of glycylglycine and Triton X-100, as previously described (Ghersi-Egea et al., 1994). The specific activity was calculated using an extinction coefficient of 9900 M^-1^.cm^-1^ for carboxy 4-nitoaniline. Glutathione*-S-*transferase activity was measured by kinetic spectrophotometry in the Cary 100 spectrophotometer using 1-chloro-2,4-dinitrobenzene (Sigma), a multispecific substrate of glutathione-*S*-transferase isoenzymes, as previously described (Ghersi-Egea et al., 2006). The specific activity was calculated using an extinction coefficient of 9600 M^-1^.cm^-1^ for the glutathione conjugate. Total protein content was measured by the method of Peterson (Peterson, 1977) using BSA to generate the standard curve.

### Statistics

Considering the number of structures (choroidal tissue, isolated microvessels, cerebral and cerebellar parenchyma), and the variable number of samples per tissue (n = 3 for isolated microvessels), the expression levels of barrier-related molecular attributes were compared among the different cerebral tissues by the non-parametric Kruskal and Wallis test corrected for multiple comparisons by the false discovery rate method of Benjamin and Hachberg (PRISM software). For clarity, only statistical differences discussed in the result section are marked on Figures 2, 4, 6. The complete set of statistic data is found in Supplementary Table S1. In all figures and in Supplementary Table S1, *, and ** indicate discoveries (statistical differences) when Q was set at 0.05, and 0.01, respectively. q Values close to significance when Q was set at 0.05 are also reported.

## Results and discussion

### Quality of isolated microvessel and choroid plexus preparations

**FIGURE 1:**
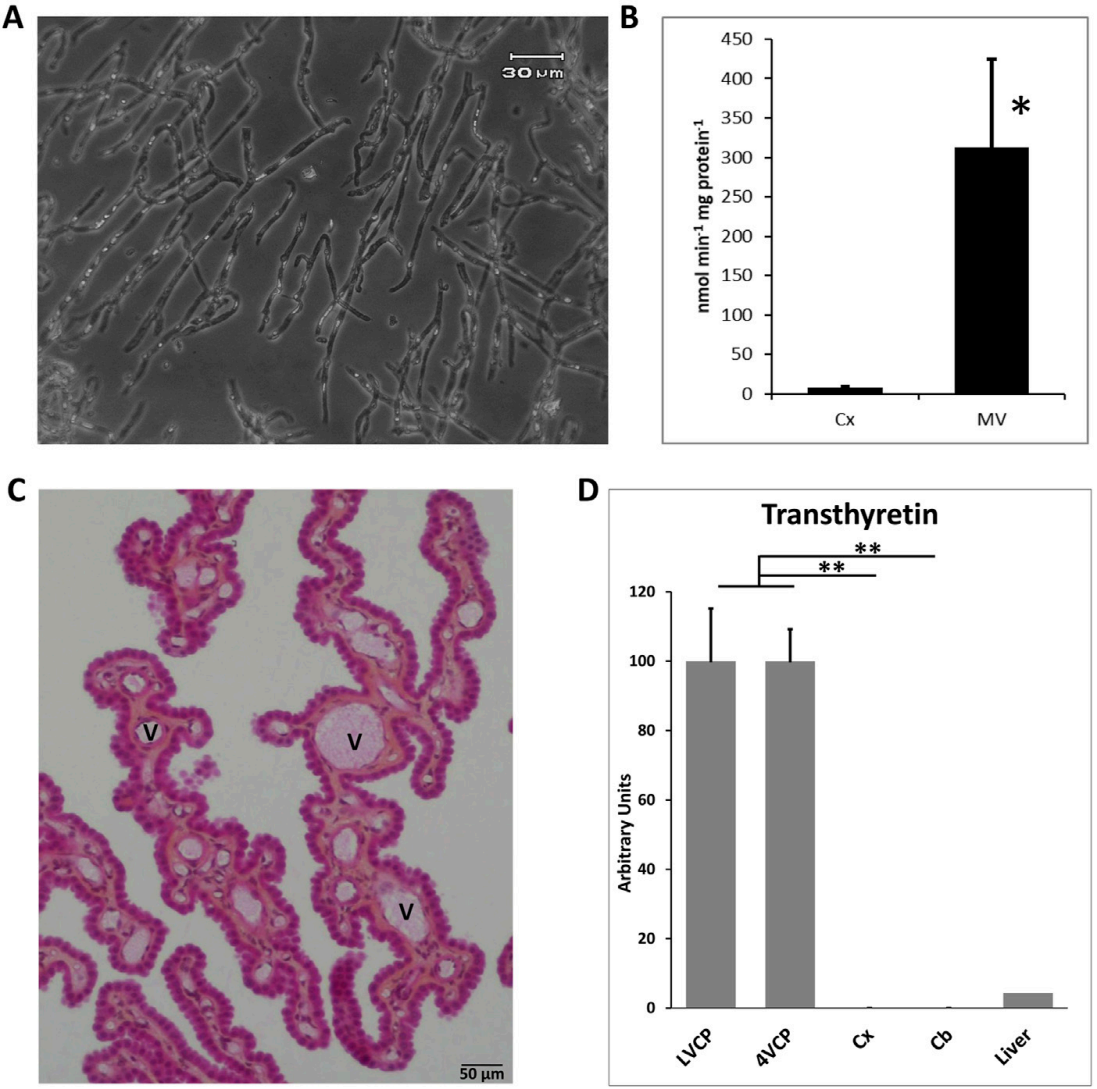
Validation of blood-brain interfaces isolated from cynomolgus monkeys. **(A)** Micrograph of isolated microvessels. The preparation contains mainly branched capillaries. **(B)** Gamma-glutamyl-transpeptidase specific activity measured in cerebral cortex and microvessel homogenates. The activity is 32- to 44-fold higher in microvessels than cerebral cortex. Mean ± SD, n = 3 preparations from two animals. **(C)** Histology of choroid plexuses. The integrity of the outer epithelial layer surrounding the vessel-containing stroma is maintained. **(D)** qPCR analysis of the choroidal epithelial marker transthyretin expression in brain tissues. A monkey liver sample was analyzed as a positive control tissue. Mean ± SD, arbitrary units. Transthyretin is exclusively expressed in choroid plexuses. Collectively these data show the purity and quality of the isolated choroidal tissue, and the absence of contaminating CP in parenchymal tissue. Scale bar: 50 µm. Abbreviations: Cx: cerebral cortex; Cb: cerebellar cortex; MV: cerebral microvessels; LVCP and 4VCP: choroid plexus of the lateral and fourth ventricle, respectively; Panel **(C)**, V: vessel.

The brain microvessel isolation technique established for the rat (Gazzin et al., 2008) was applied to the cynomolgus monkey. It produced preparations that were highly enriched in capillaries and virtually devoid of tissue microfragments (Figure 1A). The activity of γ-glutamyltransferase, a key enzyme of the glutathione cycle used as a marker of cerebral endothelial cells (Ghersi-Egea et al., 1987), was enriched on average 40 times in microvessel homogenates compared to the initial cortex homogenates (Figure 1B). Of note, activities measured in both the microvessel fraction and cerebral cortex of cynomolgus were twice those measured in rat preparations (data not shown), indicating that glutathione-dependent metabolic pathways may be especially active in cynomolgus monkey brain. Isolated microvessels also comprise pericytes as they are embedded in a common basal membrane with endothelial cells. As a result, the expression of *PDGFRβ* ^1^, a marker of pericytes, was increased 3.8 ± 0.9-fold (n = 3 p < 0.01, paired t-test) in capillaries *versus* initial whole brain tissue. In contrast, while astrocytic end-feet ghosts can remain occasionally attached to isolated capillaries, mRNA from glial origin was low in the microvessel preparations, exemplified by *GFAP* mRNA level which was only 7.1 ± 4.8% of the levels measured in corresponding whole brain tissue (n = 3, p < 0.05, paired t-test). Choroid plexuses were microdissected from the lateral and fourth ventricles of cynomolgus monkey brains. Choroid plexuses from the two locations were kept separated for the analyses. Although closely related, they may not share the same level of neuroprotective capacity. Conventional histology showed a preserved organization of the choroidal villi with a well-preserved epithelial layer surrounding the vascularized stroma (Figure 1C). Total RNA from choroidal tissue, isolated microvessels, cortical and cerebellar parenchyma were isolated, reverse transcribed and subjected to qPCR. All mRNA preparations used in this study were assessed for quality (see method). As expected, the choroidal epithelial marker transthyretin was expressed at a high level in the choroid plexuses (Figure 1D), and was undetected in cortical and cerebellar parenchyma. Three microvessel fractions were incorporated in the qRT-PCR analyses as they yielded mRNA of good quality. They were obtained from two different cortical tissue pieces of cynomolgus #7 (2 preparations) and one cortical piece of cynomolgus #8 (1 preparation). The purity of these microvessels was illustrated by the expression of the endothelial marker *PECAM-1* which was enriched 30 times in comparison to the initial cortical tissue used for capillary isolation (n = 2). These isolated cynomolgus monkey microvessel and choroid plexus preparations were therefore validated for further characterization of the molecular determinants responsible for the barrier phenotype of blood-brain interfaces.

### Composition of tight junctions at the blood-brain and blood-CSF barrier

**FIGURE 2:**
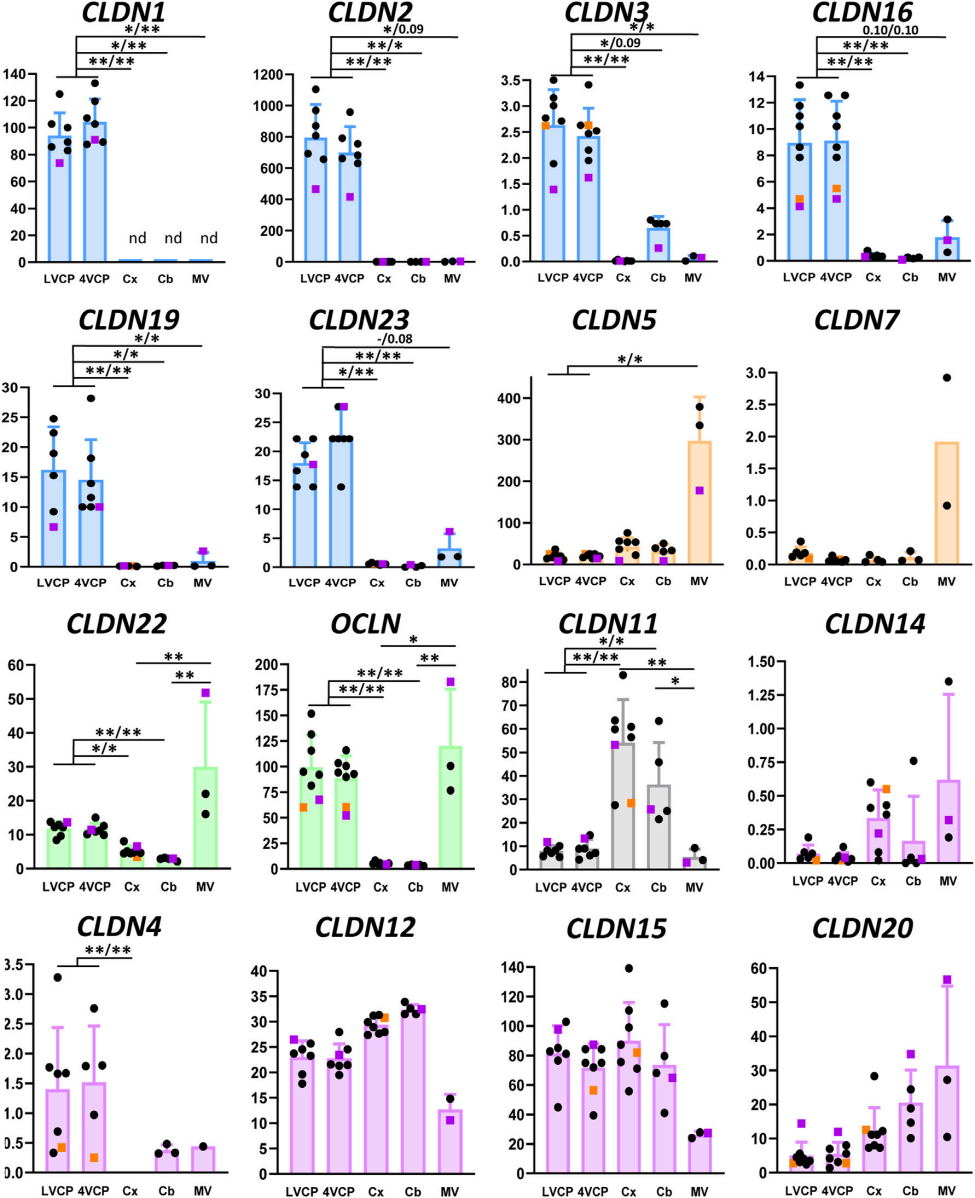
Relative levels of claudin and occludin transcripts in choroid plexuses, cerebral and cerebellar cortices and cerebral microvessels of cynomolgus monkeys. In all graphs the Y-axis is in arbitrary units, defined as level of expression relative to the housekeeping gene *DPAGT*1 and normalized to the expression level found for *CLDN1* in the LVCP sample obtained from cynomolgus monkey 2, set arbitrarily at 100 (see ‘‘Materials and methods’’ for details of calculations). Results are shown as mean ± SD, from seven to eight animals for choroid plexuses and cerebral cortex, five animals for cerebellum, and three microvessel preparations obtained from cynomolgus monkeys 7 and 8. If no standard deviation appears on the bar, then only two samples were successfully analyzed. These values were not included in the statistical analysis. Symbol colors are as follows: Pink: 9-year-old male. Orange: 5-year-old male. Black: 3.3- to 4.6-year-old females. Claudins predominantly expressed in CPs and microvessel preparations are shown in blue and orange, respectively. Claudins expressed in both brain barriers with no clear specificity are shown in green. *CLDN11* (black) is mainly expressed in brain tissue. Claudins displaying no specificity in their expression profile are shown in purple. The following claudins do not appear on the figure: *CLDN6* and *CLDN21*, which are expressed at the embryonic stage; *CLDN8* and *CLDN14*, for which a low and inconsistent expression was observed; Cldn-13, which is a murine-specific gene; *CLDN18* as it was not detected in brain preparations while readily detected in lung. *CLDN9* and *CLDN17* were detected in choroid plexuses and capillaries, respectively, but could not be accurately quantified. * and **: Selected statistically significant differences, Kruskal and Wallis test corrected for multiple comparisons, Q value set at 0.05, and 0.01, respectively. x/x indicates level of significance for LVCP and 4VCP respectively. The complete set of statistically significant differences is reported in Supplementary Table S1. Abbreviations: Cx: cerebral cortex; Cb: cerebellar cortex; LVCP and 4VCP: choroid plexus of the lateral and fourth ventricle, respectively; MV cerebral microvessels. nd: not detected.

Figure 2 shows the relative mRNA levels of claudins and occludin in cerebral microvessels and choroid plexuses. Data were obtained from juvenile and young adult females (3.3- to 4.6-year-old) with one adult and one older male (5- and 9-year-old, respectively, distinguished on the graphs) incorporated in the study. We combined all data from males and females in the statistical analyses. Of note, we could not evidence any correlation between expression and age when analysing juvenile and adult animals. The 9-year-old male animal yielded a mildly lower than average level of expression for four tight junction proteins in the choroid plexuses.

**FIGURE 3:**
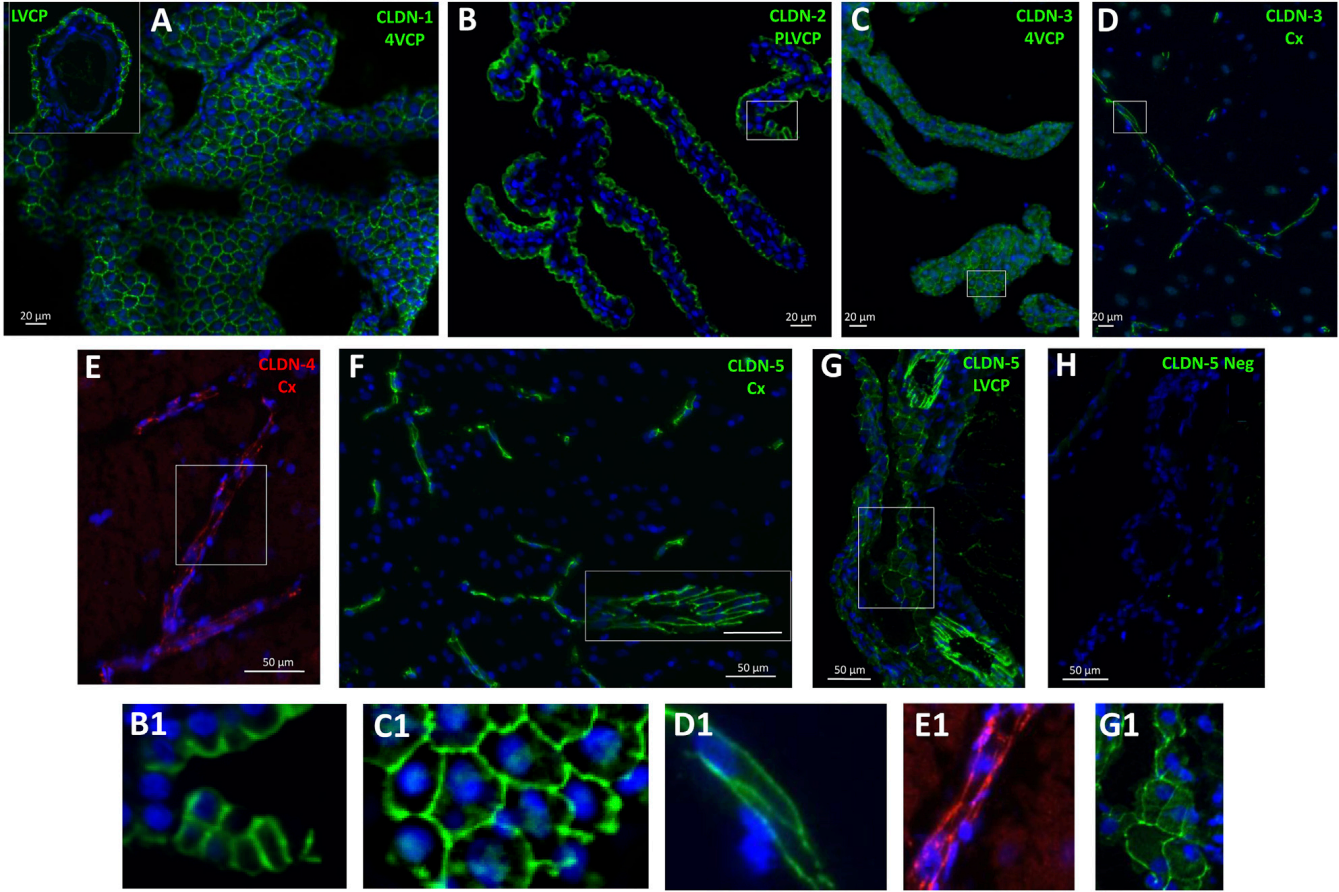
Immunohistological detection of claudins in cynomolgus monkey brain. **(A)**, CLDN-1 immunodetection reveals a strong signal associated with the epithelial cell junctions of the choroid plexuses isolated from the fourth ventricle, and lateral ventricle (**A, insert**). **(B)** and **(C)**, similarly, CLDN-2 and CLDN-3 are immunodetected at the choroidal epithelial junctions. **(D)** cell membrane-associated CLDN-3 immunoreactivity is also observed in parenchymal capillaries. **(E)** a faint, but consistent cell membrane-associated signal is observed in brain capillaries with anti-CLDN-4 antibodies. **(F)** a strong CLDN-5 immunoreactive signal is associated with the endothelial cell junctions of both small and large (insert) vessels of the brain. **(G)** CLDN-5 is also immunodetected in the endothelial junctions of the penetrating vessels within the choroidal stroma, and more faintly in cell junctions of the choroidal epithelium. **(H)** negative control obtained by omitting anti-CLDN-5 antibodies, shown at the level of the choroid plexus. B1, C1, D1, E1, G1: Enlargement of areas identified in the corresponding panels. Abbreviations: Cx: cerebral cortex; LVCP: lateral ventricle choroid plexus; 4VCP: fourth ventricle choroid plexus. Panels are also presented as single-color grey scale images without nuclei labelling in Supplementary Figure S2.

The molecular composition of tight junctions in cynomolgus monkey blood-brain interfaces shared the following features with that of tight junctions in rat and human barriers we described in Kratzer et al. (2012): *CLDN2, 1, 19* (described in rat only), and *CLDN3* (by order of abundance), which are hallmarks of the BCSFB, were specifically enriched in both LVCP and 4VCP, while *CLDN5*, characteristic of the BBB, was highly expressed and enriched in microvessels (Figure 2, blue and orange graphs). The junctional localization of these various proteins was confirmed by immunohistochemistry on monkey choroidal and parenchymal tissue (Figures 3A,B,F). *OCLN* coding for Occludin was highly expressed in both barrier fractions (Figure 2, green graph). *CLDN11* was mostly expressed in parenchymal tissue (Figure 2, black graph), as expected from its involvement in myelin sheet organization, and as we already reported for adult rat brain parenchyma (Kratzer et al., 2012).

Of note, we found that *CLDN7* and *CLDN23* mRNAs were enriched in the cynomolgus monkey microvessel and choroid plexus preparations, respectively. Cldn-7 may play a role in tight junction repair at the BBB as inferred from the recent study performed on kidney epithelial cells (Higashi et al., 2023). The relative distribution of these two claudins between the two interfaces has not been reported previously in any species. We detected mRNAs for tight junction-associated *ZO-1* and *ZO-2* in all tissues tested, with no clear enrichment in either the cerebral microvessels or choroid plexuses (Supplementary Figure S1).

This study also provided evidence for a number of differences in the composition of tight junctions in cynomolgus monkey compared to the rat (Kratzer et al., 2012).

The localization of CLDN-4, selectively expressed at the BBB in rat (Kratzer et al., 2012), was confirmed in cynomolgus monkey (Figure 3E), but the mRNA analysis revealed that in the NHP, *CLDN4* is also expressed in the choroidal tissue (Figure 2), as observed in human choroid plexuses (Rodriguez-Lorenzo et al., 2020). *CLDN16* mRNA, selectively enriched at the BBB in rat (Kratzer et al., 2012), was further enriched in choroid plexuses of cynomolgus monkey. *CLDN22* mRNA, selectively enriched at the BCSFB in rat (Kratzer et al., 2012), was also expressed at the BBB in the cynomolgus monkeys (Figure 2).

CLDN-3, selectively expressed and immunoreactive in the BCSFB in rat (Kratzer et al., 2012), was detected by immunohistochemistry at both interfaces in cynomolgus monkey (Figures 3C,D). We also observed in cynomolgus monkey additional CLDN-1 immunoreactivity in inter-endothelial junctions of cortical microvessels and larger vessels (data not shown), an observation previously reported for human (Tran et al., 2016). The expression of CLDN-3 at the BBB is debated and may vary between species (Kratzer et al., 2012; Steinemann et al., 2016; Tran et al., 2016). Given the low level of *CLDN3* mRNA measured in the monkey microvessels compared to the level in choroid plexuses, and the absence of detection of *CLDN1* mRNA in the microvessel fraction (Figure 2), these two claudins, if present at the cynomolgus monkey BBB, are probably only marginally expressed. Although anti-claudin antibodies are claudin-specific, a cross-reactivity between CLDN-1 or CLDN-3 and other tight junction proteins at the cerebral capillaries may occur, as reported for Cldn-3 in mice (Castro Dias et al., 2019). The parenchymal expression of *CLDN3* restricted to the cerebellum remains also to be understood (Figure 2). Yet, a potential contamination of the cerebellar tissues by appended 4VCP fragments was ruled out by the complete absence of transthyretin expression in these preparations. A robust CLDN-5 immunoreactivity was observed in cynomolgus monkey, not only at the BBB and in endothelial junctions of large choroidal vessels as observed previously in rats (Kratzer et al., 2012), but also in junctions between choroidal epithelial cells (Figures 3G,H). The latter labelling has not been observed in the rat choroidal tissue, but has been observed in the choroidal epithelium during human development (Mollgard et al., 2017; Virag et al., 2017). Using RNA sequencing of adult human CPs, Rodriguez-Lorenzo and colleagues showed a substantial *CLDN5* expression in this tissue (Rodriguez-Lorenzo et al., 2020), but did not assess CLDN-5 localization in their study. Collectively, these observations call for a clear understanding of the exact contribution of the numerous tightening (e.g., CLDN-5) and pore-forming (e.g., CLDN-2) claudins present at the BCSFB in setting the fence function of this interface. Differences in tight junction proteins between species may change the function of epithelial junctions and subsequently modify selectively blood to CSF permeation.

Taken together, our data indicate that the blood-brain and blood-CSF barriers in cynomolgus monkey share more analogy with the human barriers than the rodent barriers with regard to the protein composition of their tight junctions.

### Expression of drug efflux ABC transporters at the blood-brain and blood-CSF barriers

#### ABCB1 and ABCG2

**FIGURE 4:**
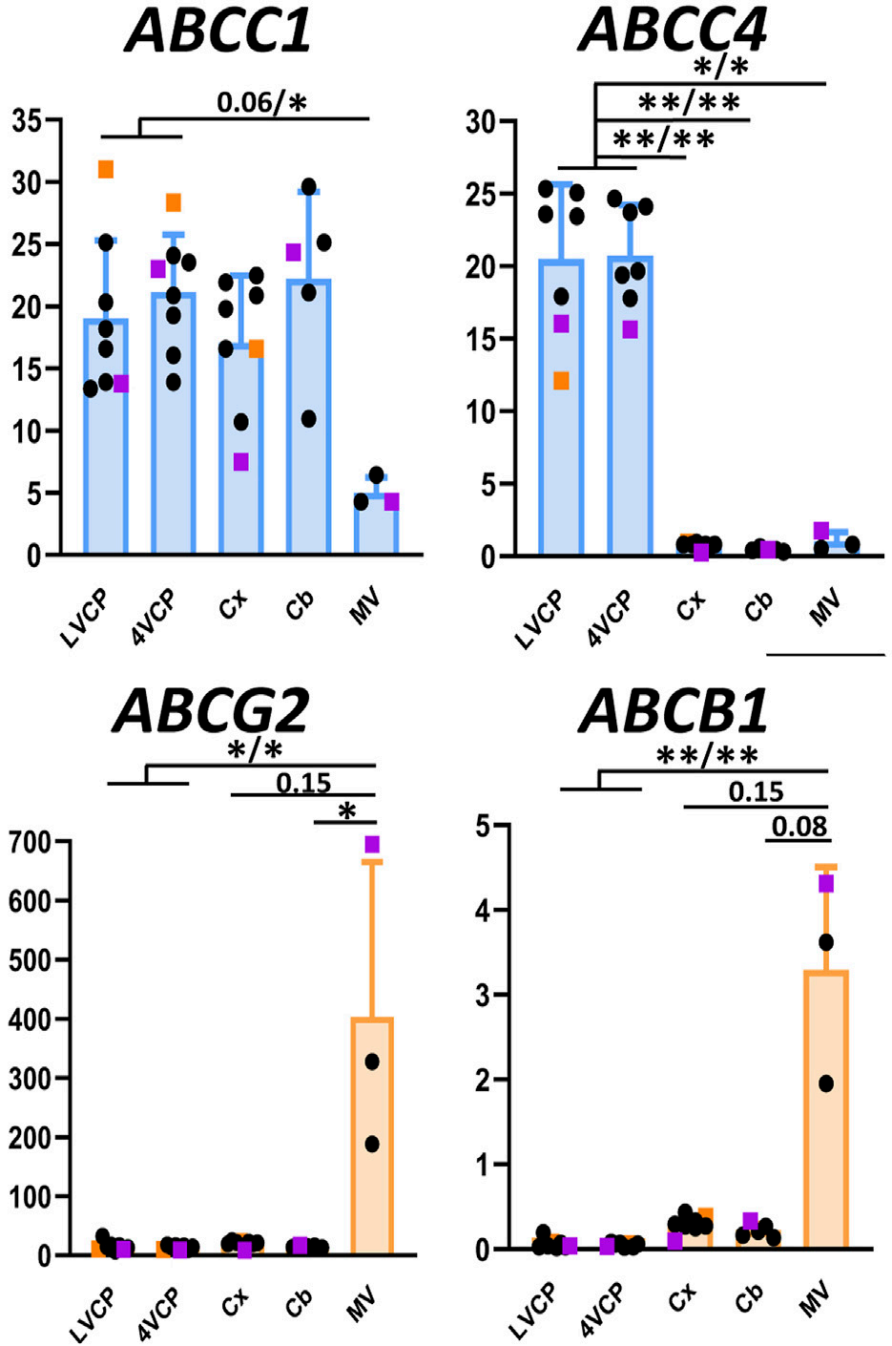
Relative levels of multispecific ABC efflux transporter transcripts in choroid plexuses, cerebral cortices and cerebral microvessels of cynomolgus monkeys. The Y-axis is in arbitrary units defined as level of expression relative to the housekeeping gene *DPAGT1* and normalized to the expression level found for *CLDN1* in the LVCP sample obtained from cynomolgus monkey 2, set arbitrarily at 100 (Figure 2). Results are shown as mean ± SD from seven to eight animals for choroid plexuses and cerebral cortex, five animals for cerebellum and three microvessel preparations obtained from cynomolgus monkeys seven and 8. Genes predominantly expressed in CPs and microvessel preparations are shown in blue and orange, respectively. Abbreviations, symbol colors and statistical analysis are as in Figure 2.

**FIGURE 5:**
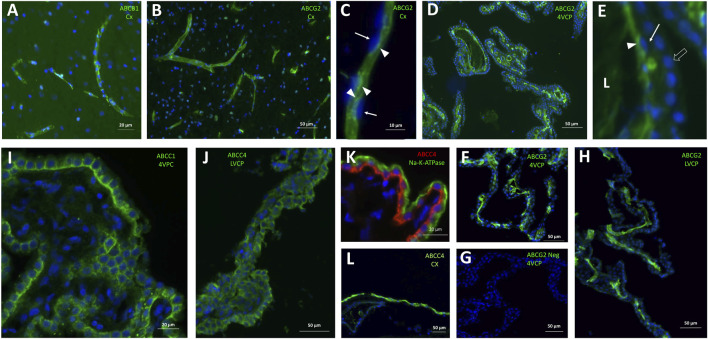
Immunohistological detection of mutispecific ABC transporters in cynomolgus monkey brain. **(A)** ABCB1 is immunodetected at capillaries within the brain parenchyma. **(B, C)** ABCG2 immunoreactivity is strong in all brain microvessels **(B)**, and is associated with the luminal membrane of endothelial cells **(C)**. Its luminal localization is assessed in relation to the endothelial nucleus staining (arrows in **(C)**. **(D-H)** ABCG2 immunoreactivity is also observed in both large and small vessels within the choroid plexuses isolated from the fourth ventricle (D,F, two different animals), and lateral ventricles **(H)**. In panel E (enlargement from panel D), the choroidal vessel endothelium can be distinguished from the epithelium identifiable by the row of round nuclei (arrowhead). Endothelial ABCG2 localization appears luminal, as shown by its staining relative to the endothelial nuclei (arrows). A negative control of immunolabeling shown in **(F)**, obtained by omission of the primary antibody is shown in **(G)**. **(I, J)** both ABCC1 **(I)** and ABCC4 **(J)** antibodies stain the basolateral membrane of the epithelium in choroid plexuses isolated from all ventricles. **(K)** this basolateral localization is further evidenced by co-labelling with Na-K-ATPase, a marker of the apical membrane of the choroidal epithelium. **(L)**, a strong ABCC4 immunoreactivity is also observed on the meningeal, presumably arachnoid, membrane. L: Lumen of the choroidal vessel. Other abbreviation as in Figure 3. Panels are also presented as single-color grey scale images without nuclei labelling, or using blue/yellow pair of colors, in Supplementary Figure S3.

Among ABC transporters involved in the efflux of drugs, both *ABCB1* and *ABCG2* genes were expressed at much higher levels in monkey brain microvessel preparations in comparison to the choroid plexuses (Figure 4, orange graphs), as described in all other mammalian species so far. Immunoreactivity of the corresponding proteins in brain vessels demonstrated a luminal localization (Figures 5A–C), as already observed in rat and human by different groups (Dauchy et al., 2008; Gazzin et al., 2008). The level of *ABCG2* mRNA was substantially higher than that of *ABCB1* in cynomolgus monkey brain microvessels (Figure 4, note scale differences). This superior level of *ABCG2* expression was already observed in human, albeit to a lesser degree (Dauchy et al., 2008). Proteomic analyses performed on microvessels isolated from marmoset (Hoshi et al., 2013) and cynomolgus monkey (Ito et al., 2011) brains indicated that the difference in transcript levels of the two genes we observed in monkey is reflected at the protein level. In contrast, protein levels of ABCB1 seem higher than those of ABCG2 in rats (Ito et al., 2011; Hoshi et al., 2013; Omori et al., 2020). Some proteomic studies highlighted a higher level of ABCG2 protein as compared to ABCB1 protein in microvessels isolated from human brain (Shawahna et al., 2011; Billington et al., 2019; Bao et al., 2020; Storelli et al., 2021), while other studies did not confirm this difference (Uchida et al., 2011; Al-Majdoub et al., 2019). A larger implication of ABCG2 over ABCB1 in controlling cerebral efflux processes at the human BBB would have pharmacological and pathophysiological significance as ABCG2 and ABCB1 have different, although partially overlapping, substrate specificities (Choi and Yu, 2014), and different substrate affinities (Hegedus et al., 2009). For instance, ABCB1 rather than ABCG2 is likely involved in amyloid β peptide efflux from the brain (Wolf et al., 2012).

In the choroidal tissue of cynomolgus monkeys, we found a very low expression of *ABCB1* (Figure 4). *ABCB1* mRNA was identified in human CP (Rodriguez-Lorenzo et al., 2020), but protein levels have been shown to be very low in both rat and human choroidal tissues (Gazzin et al., 2008). Accordingly, we could not immunodetect ABCB1 in either epithelial or endothelial cells of monkey CP. *ABCG2* transcripts were identified in monkey choroid plexus tissues, albeit at low levels by comparison to the level measured in isolated microvessels (Figure 4). In human CP, *ABCG2* mRNA was also identified, at low level similar to that of *ABCB1* (Rodriguez-Lorenzo et al., 2020). Previous studies reported low and developmentally regulated *ABCG2* mRNA levels in rat choroidal tissue (Ek et al., 2010; Kratzer et al., 2013), undetectable *ABCG2* transcripts in developing and adult mouse choroidal tissue (Tachikawa et al., 2005), and low but sizable ABCG2 protein levels in membrane fractions isolated from adult rat choroidal tissue (Uchida et al., 2015). Our immunohistological analysis of ABCG2 in monkey choroid plexus failed to detect the transporter in the outer epithelial layer, but located it in the vessel loops irrigating the choroidal villi (Figures 5D–G), a localization also observed in human (Mollgard et al., 2017) and mice (Orford et al., 2009). As epithelial cells constitute a large proportion of the choroidal tissue, this restricted endothelial localization of ABCG2 explains why the expression level we observed in the choroidal tissue as a whole is lower than in isolated microvessels (Figure 4), despite the strong immunohistochemical signal observed in both brain and choroidal endothelia. The apparent luminal localization of ABCG2 (Figure 5E) indicates a transport directionality towards blood, which suggests that the choroidal endothelium is less permissive than previously thought to ABCG2 substrates. Previously published attempts to locate ABCG2 at the choroidal epithelium itself by immunohistochemical analyses generally concluded to the absence of the transporter at this location in adult rat and human. In mouse, the data were conflicting, possibly due to immunohistochemical methodology issues (reviewed in (Matsumoto et al., 2015; Strazielle and Ghersi-Egea, 2015). These published data, coupled to our results showing an immunohistochemical signal restricted to the endothelium in adult cynomolgus monkey, indicate that ABCG2-dependent drug transport at the choroidal epithelium, if existing, is likely limited to the early period of brain development.

#### ABCC1

The *ABCC1* gene was expressed at a higher level in cynomolgus monkey choroid plexuses than in cerebral microvessels (Figure 4, blue graph), and the protein was immunodetected at the basolateral membrane of the choroidal epithelium (Figure 5I). A similar pattern was previously reported for ABCC1 protein in rat and human (Gazzin et al., 2008). In contrast to the consensus on ABCC1 expression and function at the BCSFB, the expression and functional relevance of ABCC1 at the BBB is still debated. Our present data indicate that in cynomolgus monkey, ABCC1 is mainly a blood-CSF rather than a blood-brain barrier component. The expression level of *ABCC1* was even higher in cortical and cerebellar tissues than in capillary preparations (Figure 4), a difference in distribution observed neither in rat for the protein (Gazzin et al., 2008), nor in human for mRNA (Dauchy et al., 2008). This may indicate that cells forming the neuropil, especially astrocytes (Gennuso et al., 2004), are an important source of ABCC1 in the brain of cynomolgus monkeys. In line with this result, a vesicular labelling was observed in a large proportion of cortical parenchymal cells in cynomolgus monkey brains by immunofluorescence using an anti-ABCC1 antibody (data not shown).

#### ABCC4

*ABCC4* gene expression was found to be largely enriched in the BCSFB in cynomolgus monkey (Figure 4, blue graph). At this site ABCC4 was exclusively located at the basolateral, blood-facing membrane of the choroidal epithelium (Figure 5J), clearly distinguishable from the apical membrane identified by Na-K-ATPase labelling (Figure 5K). This strictly basolateral localization was previously described in other species (Leggas et al., 2004; Strazielle et al., 2005). We could not detect ABCC4 protein in cynomolgus monkey cerebral cortex by immunohistochemistry, while vessels were labelled in rat sections (data not shown). ABCC4 has been previously localized at both barriers in rodents, where it actively prevented the antitumoral drug topotecan to accumulate in the brain and CSF (Leggas et al., 2004). In this latter study, the increase in topotecan concentration between wild type and knockout mice was however higher in CSF than in brain tissue, suggesting that ABCC4 is especially active at the BCSFB. In human, no *ABCC4* mRNA enrichment was observed in cerebral capillaries compared to the cortical tissue, by contrast to the strong enrichment observed for *ABCB1* and *ABCG2* (Dauchy et al., 2008). Another study showed both at the mRNA and protein level that ABCC4 enrichment in brain capillaries was more prominent in rat than in human (Warren et al., 2009). Thus, the higher expression of ABCC4 at the BCSFB compared to the BBB represents another feature shared by human and non-human primates, that is not observed in rodents. Besides drugs, ABCC4 transports prostaglandin E2 (PGE2), (Reid et al., 2003), a neuroinflammatory modulator which is not inactivated within the brain (Alix et al., 2008), but removed by transport at blood-brain interfaces (Khuth et al., 2005; Akanuma et al., 2010). The species differences in the localisation of ABCC4 at blood-brain interfaces therefore highlight choroid plexuses as the main site for PGE2 signal termination in human and non-human primates. Of note, a strong ABCC4 immunohistochemical signal was also observed at the arachnoid membrane separating the subarachnoid CSF from the dura matter (Figure 5L). Immunohistochemistry data have to be interpreted with caution in these leptomeningeal areas, owing to the frequent nonspecific border effects associated with immunochemical signals at outer brain membranes, and further investigations are needed to confirm the localization of efflux transporters at the arachnoid in primates.

The ABCC1 and/or ABCC4 transporters are likely to be the active carriers exporting into blood the conjugates formed within the choroidal epithelium by the glutathione-S-transferases, thus participating in a cerebral mechanism of toxicant inactivation described in rat and human (Ghersi-Egea et al., 2006; Kratzer et al., 2018). We measured the overall conjugating activity to glutathione in monkey tissues using 1-chloro-2,4-dinitrobenzene as a prototypic substrate. The specific enzymatic activities ranged from 114 to 175 nmol min^-1^. mg prot^-1^ and 124–273 nmol min^-1^. mg prot^-1^ in LVCP and 4VCP, respectively (ranges obtained for four animals, two males and two females, with no sizeable sex difference). These choroidal activities were 3–5 times higher than those measured in the cerebral cortex (42-58 nmol min^-1^. mg prot^-1^, p < 0.01 and 0.05 for LVCP and 4VCP, respectively, two-tailed Student’s t-test for unequal variance). Thus, the coupled metabolic/efflux transport process, which acts in the rat BCSFB as a mechanism of neuroprotection towards toxicants should also be functional in the cynomolgus monkey choroidal barrier.

### Expression of other drug efflux transporters at the blood-brain and blood-CSF barriers

Besides ABC efflux transporters, various carriers of the SLC superfamily can also influence the cerebral bio-availability of drugs, toxic compounds, and biologically active metabolites. They include members of the *SLCO*, *SLC22* and *SLC47* families, which display distinct substrate specificity profiles.

**FIGURE 6:**
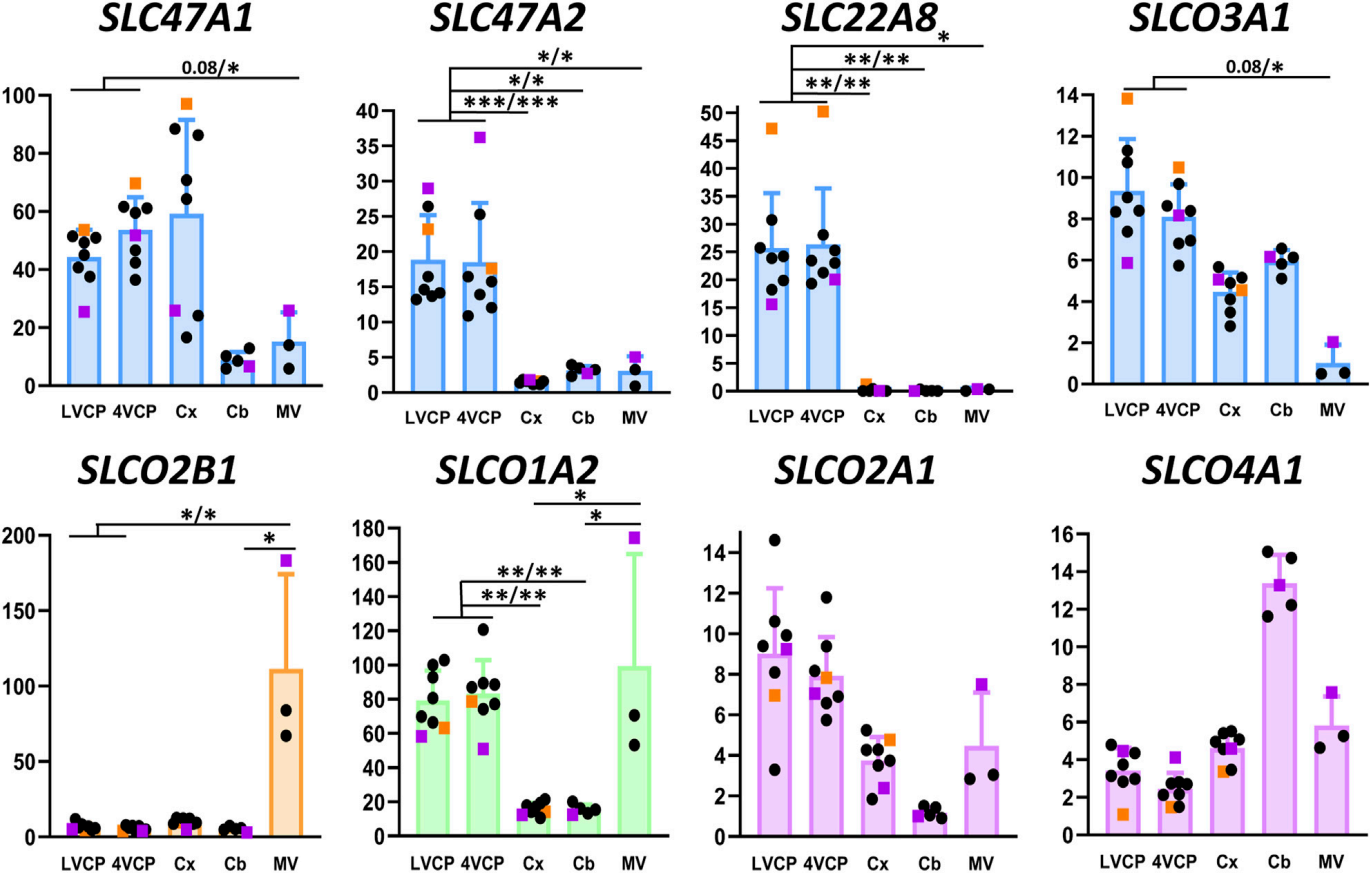
Relative levels of multispecific SLC efflux transporter transcripts in choroid plexuses, cerebral cortices and cerebral microvessels of cynomolgus monkeys. The Y-axis is in arbitrary units defined as level of expression relative to the housekeeping gene Dpagt1 and normalized to the expression level found for *SLCO1A2* in the LVCP sample obtained from cynomolgus monkey 2, set arbitrarily at 100. Results are shown as mean ± SD, from eight animals for choroid plexuses and cerebral cortex, five animals for cerebellum and three microvessel preparations obtained from cynomolgus monkeys seven and 8. Symbol color, color codes, abbreviations and statistical analysis are as in Figure 2.

The present work has focused among all *SLCO* carriers, on the ubiquitous members *SLCO3A1* and *SLCO4A1*, and the BBB-specific members (within the brain) *SLCO2B1* and *SLCO1A2* (Bronger et al., 2005; Roth et al., 2012; Ronaldson and Davis, 2013). The OATP proteins encoded by these four genes accept various xenobiotics as substrates, such as antibiotics, endothelin A receptor antagonists, beta-blockers, antiretroviral and antineoplastic agents. Endogenous compounds such as steroids, thyroid hormones, prostaglandins are also transported by these carriers. The *SLCO2A1*-encoded OATP carrier more specifically transports prostaglandins (for reviews on substrate selectivity of these transporters, see Roth et al., 2012; Ronaldson and Davis, 2013). The expression profile of these transporters in brain barriers and parenchyma of cynomolgus is described in Figure 6. The robust expression of S*LCO2B1* and *SLCO1A2* genes in the non-human primate BBB, in line with data obtained in human tissues, stresses the probable influence of these two proteins on cerebral drug bioavailability (Morris et al., 2017; Schafer et al., 2020; Kinzi et al., 2021). Both messengers were enriched in microvessels compared to the cortex and cerebellum, corroborating the high microvessel-to-tissue ratios previously reported for mRNA levels in human brain (Gao et al., 2000; Geier et al., 2013; Suhy et al., 2017). Immunohistochemical and quantitative proteomic analyses reported by other groups consistently detected *SLCO2B1*-encoded protein in microvessels isolated from human brain, while *SLCO1A2*-encoded protein was not always detected (Uchida et al., 2011; Gao et al., 2015; Billington et al., 2019; Bao et al., 2020; Schafer et al., 2020). In cynomolgus choroid plexuses, we found high mRNA levels of *SLCO1A2*, but not *SLCO2B1* (Figure 6). This is in contrast with human transcriptomic data showing a higher expression of *SLCO2B1* as compared to *SLCO1A2* in this tissue (Rodriguez-Lorenzo et al., 2020), and with a proteomic study that failed to detect *SLCO1A2*-encoded protein in one specimen of human choroid plexus tissue (Uchida et al., 2015). The expression of *SLCO2A1* and *SLCO4A1* genes was low in all cynomolgus tissues we investigated, with no apparent specificity for either barrier. In contrast, *SLCO3A1* expression was higher in choroid plexuses than in microvessels isolated from monkey brain, and intermediate in parenchyma (Figure 6). In line with this pattern, immunohistochemical analysis of human brain sections has shown that the two splice variants of human OATO3A1 were associated with the membrane of choroid plexus epithelial cells, and were detected in neural cells of the frontal cortex (Huber et al., 2007). Comparing the expression of these *SLCO* genes between primate and rodent is strongly impeded by the low homology of sequences between these species, which is characteristic of the *SLCO* family (Hagenbuch and Meier, 2004). Overall, our data suggest that the expression of *SLCO* genes of interest in the primate BBB resembles that observed in human, and further provide a detailed pattern of expression for these genes at the BCSFB. Of note, OATP-dependent transport processes can be bidirectional, and the membrane localization of most OATP proteins is unknown in the primate blood-brain and blood-CSF barriers. Whether these transporters influence positively or negatively the cerebral penetration of their substrates remains a poorly understood field of research in primates.

The organic anion transporter SLC22A8 has been shown to play an important role as an efflux transporter for many drugs including antivirals and antibiotics, and for endogenous compounds such as steroid derivatives at both brain barriers in rats and mice (Strazielle et al., 2003; Sykes et al., 2004; Ose et al., 2009). We found a high expression of this gene in choroid plexuses isolated from monkey brain, while its expression was barely detectable in other tissues investigated including microvessels (Figure 6). Accordingly, the corresponding protein has not been detected in a quantitative proteomic analysis of microvessels isolated from cynomolgus monkey brains (Ito et al., 2011). In human brain microvessels, the protein was identified in only one study, albeit at a low level compared to ABC transporters (Al-Majdoub et al., 2019), and was not detected in several other targeted proteomic analyses (Shawahna et al., 2011; Uchida et al., 2011; Billington et al., 2019; Bao et al., 2020). *SLC22A8* expression level was higher than that of *ABCC1* in human CP (Rodriguez-Lorenzo et al., 2020), and the protein was present at a level similar to that of ABCC1 in one sample of human choroid plexus (Uchida et al., 2015). Collectively, these data suggest that, with respect to the relative expression of *SC22A8* between the two barriers, a higher similarity of SLC22A8-dependent efflux processes exists between non-human and human primates than between primates and rodents.

Finally, we investigated carriers of the *SLC47* family for which differences in expression have been reported between human and non-primate species. The protein multidrug and toxin exclusion (MATE)1 encoded by *SLC47A1* was detected in substantial amounts by absolute targeted proteomic in human choroid plexuses, but was under the limit of quantification in rat tissue (Uchida et al., 2015). In cynomolgus monkey, we found that *SLC47A1* and *SLC47A2* levels of expression were both higher in choroid plexuses than in brain microvessels (Figure 6), a finding that is consistent with the modest expression, if any, of *SLC47A1* in human brain microvessels (Shawahna et al., 2011). We also detected *SLC47A1* mRNA in monkey cortex and cerebellum, while *SLC47A2* expression was more specific of the choroid plexuses (Figure 6). Fairly high levels of message for both transporters were also detected in human choroid plexuses (Rodriguez-Lorenzo et al., 2020). Yet, the protein MATE2-K, the main variant isoform encoded by *SLC47A2* in kidney, was under the limit of quantification in human choroid plexus (Uchida et al., 2015). The proteins encoded by *SLC47* genes, MATE1 and MATE2-K are proton-coupled antiporters for small organic cations. They accept endogenous and exogenous cations as substrates, including a large range of therapeutic agents, natural products such as flavonoids, and potentially toxic molecules such as creatinine, some neurosteroids, or else methylphenylpyridinium (Motohashi and Inui, 2013; Nies et al., 2016). The substrate specificity of other SLC47A2 variant isoforms is currently unknow except for tetraethylammonium (Nies et al., 2016). More data on the protein isoforms actually present in the BCSFB, their membrane localization, and their substrate specificities are needed before their involvement in the clearance of organic cations from the CNS can be fully clarified in primate.

## Conclusions

A summary of the main similarities and differences between cynomolgus monkey, rat, and human in the molecular attributes setting neuroprotective functions at blood-brain interfaces is provided in Table 4. While the main tight junction and drug transport characteristics of brain barriers are shared between mammalian species, the cynomolgus monkey shares with human some differences in the gene expression and barrier distribution of selected claudins and efflux transporters in comparison to rodents. This needs to be kept in mind when designing and interpreting the results of cerebral toxicological and drug delivery studies. Our knowledge of these species differences should also clarify the pathogenic mechanisms which involve changes in the integrity of tight junctions or efflux transporters, such as met in neuroinflammatory and neurodegenerative diseases. An alteration of tight junctions may impact immune cell invasion across an inflamed cerebral endothelium or choroidal epithelium. A dysfunction of efflux transporters can lead to eicosanoid and amyloid β peptide accumulation in the brain. Finally, our analysis provides the first image of drug carriers expression at the primate BCSFB, a mandatory step toward a better control of cerebral drug delivery *via* the BCSFB.

**TABLE 4:** Summary of the main similarities and differences observed among species concerning the molecular attributes setting neuroprotective functions at blood-brain interfaces.

Tight junction proteins	Monkey	Rat	Human
CLDN-1, -2, -3	- CPs (epithelium) - CLDN-1 and -3 Immunoreactivity in MVs despite undetected CLDN-1 and low CLDN-3 expression	- CPs (epithelium)	- CPs (epithelium) - CLDN-1 Immunoreactivity in MVs
CLDN-19	- CPs	- CPs (epithelium)	Unknown
CLDN-4	- MVs - CPs	- MVs only	Unknown
CLDN-5	- MVs and larger choroidal vessels - Immunoreactivity in CP epithelium	- MVs and larger choroidal vessels	- MVs and larger choroidal vessels - Immunoreactivity in CP epithelium
CLDN-11	- Brain parenchyma	- Brain parenchyma	- Brain parenchyma
CLDN-16	- Enriched in CPs	- Enriched in MVs	?
CLDN-22	- CPs - Enriched in MVs	- CPs	- CPs - localization in MVs unknown
OCLN	- both CPs and MVs	- both CPs and MVs	- both CPs and MVs
Transporters			
ABCB1 and ABCG2	- MVs - ABCG2>>ABCB1 in MVs	- MVs - ABCB1>ABCG2 in MVs	- MVs - Close levels of ABCG2 and ABCB1 in MVs
ABCG2	- Low overall expression in CPs, with a localization in choroidal endothelial loops	- Low overall expression in CPs	- Low overall expression in CPs, with a localization in choroidal endothelial loops
ABCC1	- CPs (epithelium) - High expression in brain parenchyma	- CPs (epithelium)	- CPs (epithelium)
ABCC4	- CPs (epithelium) - Not detected in MVs	- CPs (epithelium) -MVs	- CPs (epithelium) - No or low expression in MVs
SLCO2B1	- MVs only	Homology between rodent and primate SLCO genes is poor	- MVs -CPs
SLCO1A2	- MVs -CPs		- MVs - (CPs)
SLCO3A1	- CPs		- CPs
SLC22A8	- CPs only	- CPs - MVs	- CPs (no or low expression in MVs)
SLC47A1 and SLC47A2	- CPs - Isoforms of SLC47 expressed in the CPs may differ from that of human	-Undetected in CPs	- CPs

The summary is based on both expression and localization studies, see text for detailed analysis and for references related to human and rat data. The information reported for monkey that has not previously been reported in any other species is not listed in the table.

## Data Availability

The original contributions presented in the study are included in the article/Supplementary Material, further inquiries can be directed to the corresponding author.

## Footnotes

1 In this section gene names are italicized when specifically referring to mRNA levels.

## Abbreviations

4VCP, fourth ventricle choroid plexus; ABC, ATP-binding cassette; BBB, blood-brain barrier; BCSFB, blood-cerebrospinal fluid barrier; BSA, bovine serum albumin; CNS, central nervous system; CP, choroid plexus; Ct, cycle threshold; CSF, cerebrospinal fluid; KR, Krebs-Ringer buffer; LVCP, lateral ventricle choroid plexus; MATE, multidrug and toxin exclusion; NHP, non-human primates; OATP, organic anion-transporting polypeptide; PBS, phosphate buffer saline; PGE2, prostaglandin E2; RT-qPCR, real time quantitative polymerase chain reaction; SLC, solute carriers.
