# Supplementary figures and images for: Molecular determinants of neuroprotection in blood-brain interfaces of the cynomolgus monkey
Source: Front Pharmacol. 2025 Mar 12;16:1523819. doi: 10.3389/fphar.2025.1523819 (PMC11936797; doi:10.3389/fphar.2025.1523819)

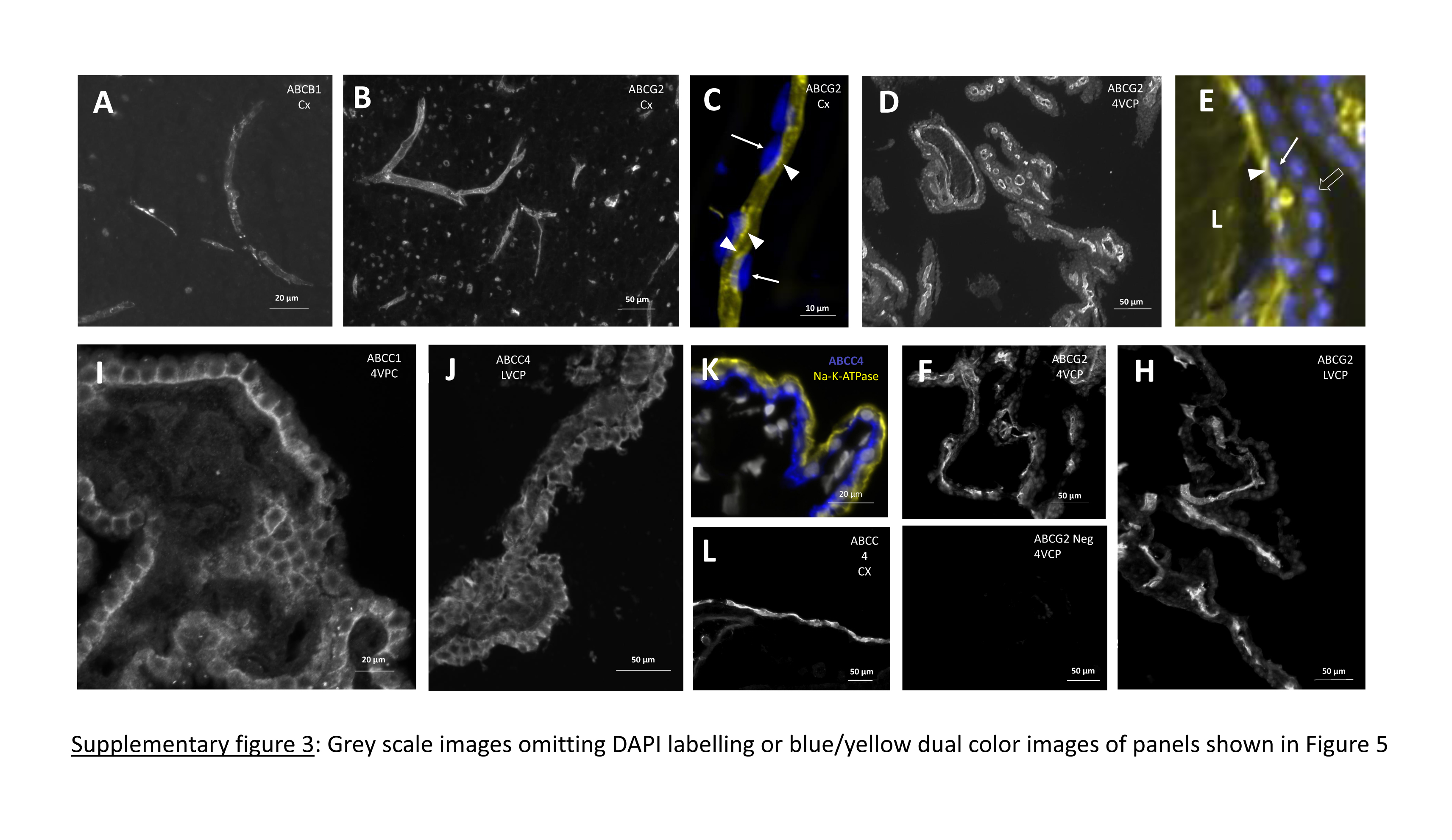

Supplement: Supplementary file 1 [file Image3.jpeg]

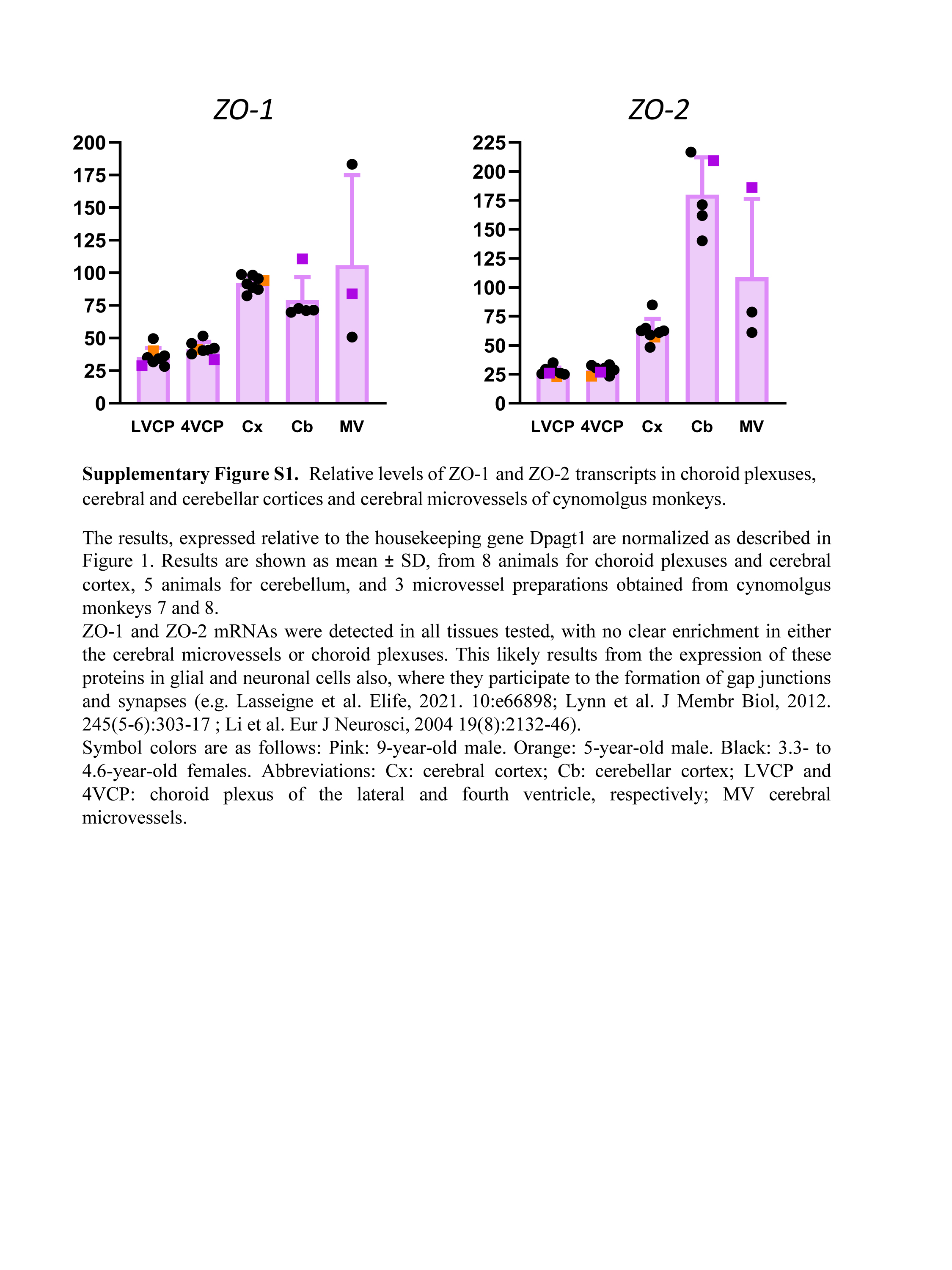

Supplement: Supplementary file 3 [file Image1.jpeg]

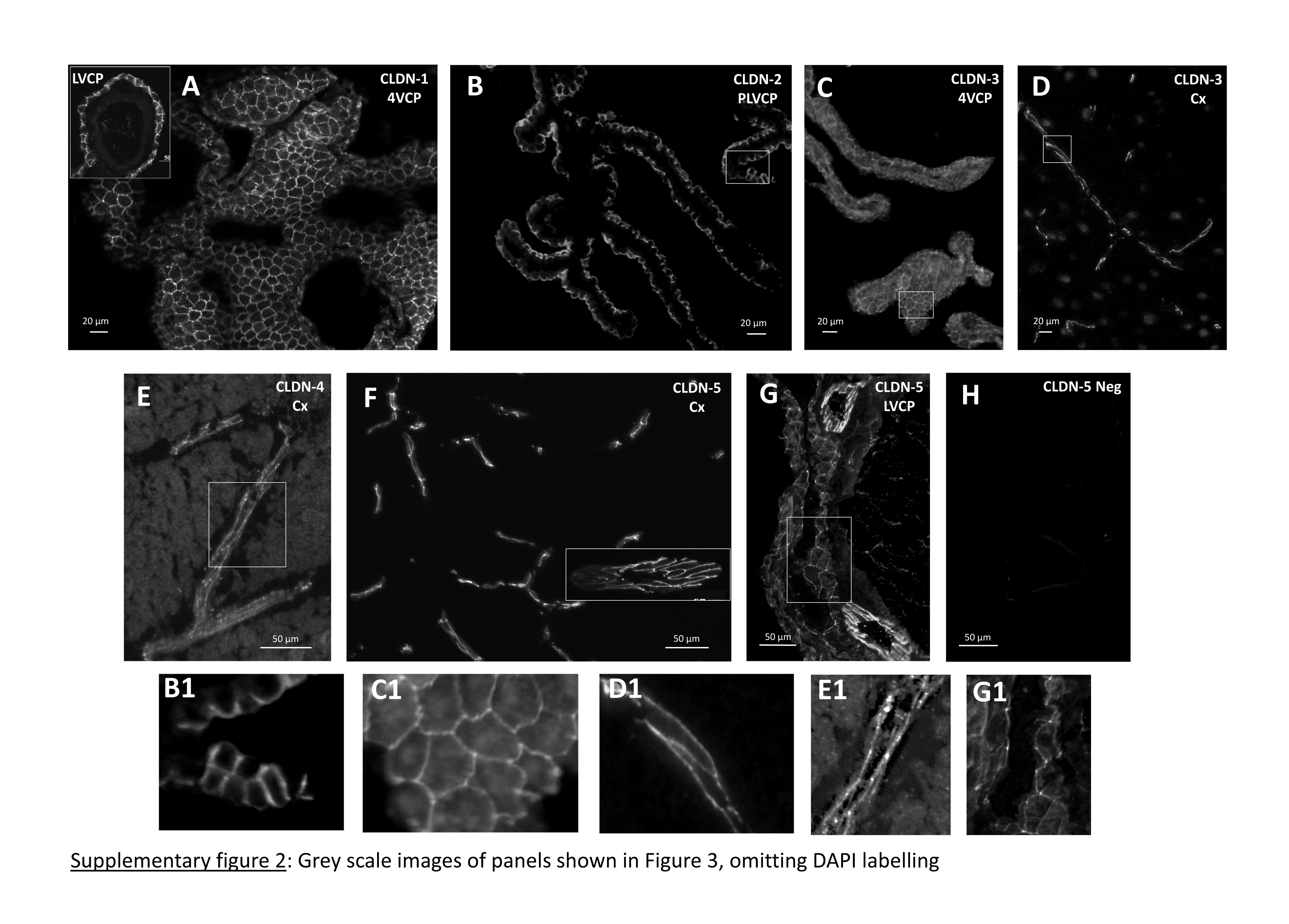

Supplement: Supplementary file 4 [file Image2.jpeg]
