# Supplementary material for: Molecular determinants of neuroprotection in blood-brain interfaces of the cynomolgus monkey
Source: Front Pharmacol. 2025 Mar 12;16:1523819. doi: 10.3389/fphar.2025.1523819 (PMC11936797; doi:10.3389/fphar.2025.1523819)
Supplement: Supplementary file 2 [file Table1.pdf]

CLDN1	LVCP	4VCP	Cx	Cb	MV
LVCP		-	**	**	*
4VCP			**	**	**
Cx				-	-
Cb					-

CLDN2	LVCP	4VCP	Cx	Cb	MV
LVCP		-	**	**	*
4VCP			**	*	0,0948
Cx				-	-
Cb					-

CLDN3	LVCP	4VCP	Cx	Cb	MV
LVCP		-	***	*	*
4VCP			***	0,0878	*
Cx				-	-
Cb					-

CLDN4	LVCP	4VCP	Cx	Cb	(MV)
LVCP		-	**	-	-
4VCP			**	-	-
Cx				-	-
Cb					-

CLDN5	LVCP	4VCP	Cx	Cb	MV
LVCP		-	0,0695	-	*
4VCP			0,086	-	*
Cx				-	0,1634
Cb					0,0959

CLDN7	LVCP	4VCP	Cx	Cb	(MV)
LVCP		-	0,0733	-	
4VCP			-	-	0,0534
Cx				-	0,0534
Cb					

CLDN11	LVCP	4VCP	Cx	Cb	MV
LVCP		-	**	*	-
4VCP			**	*	-
Cx				-	**
Cb					*

CLDN12	LVCP	4VCP	Cx	Cb	(MV)
LVCP		-	*	**	0.1082
4VCP			*	**	0.1288
Cx				-	**
Cb					**

CLDN14	LVCP	4VCP	Cx	Cb	MV
LVCP		-	-	-	-
4VCP			0.0796	-	0.0796
Cx				0.0796	
Cb					0.0796

CLDN15	LVCP	4VCP	Cx	Cb	MV
LVCP		-	-	-	*
4VCP			-	-	0.1072
Cx				-	*
Cb					-

CLDN16	LVCP	4VCP	Cx	Cb	MV
LVCP		-	**	**	0.105
4VCP			**	**	0.105
Cx					
Cb					

CLDN19	LVCP	4VCP	Cx	Cb	MV
LVCP		-	**	*	*
4VCP			**	*	*
Cx					
Cb					

CLDN20	LVCP	4VCP	Cx	Cb	MV
LVCP		-	**	**	**
4VCP			**	**	**
Cx					
Cb					

CLDN22	LVCP	4VCP	Cx	Cb	MV
LVCP		-	*	**	
4VCP			*	**	
Cx					**
Cb					***

CLDN23	LVCP	4VCP	Cx	Cb	MV
LVCP		-	*	**	
4VCP			**	***	0.0769
Cx					
Cb					

Occludin	LVCP	4VCP	Cx	Cb	MV
LVCP		-	**	**	-
4VCP			**	**	-
Cx					*
Cb					**

ABCG2	LVCP	4VCP	Cx	Cb	MV
LVCP		-	0,0671	-	*
4VCP			*	-	*
Cx				-	0,1459
Cb					*

ABCB1	LVCP	4VCP	Cx	Cb	MV
LVCP		-	**	*	**
4VCP			**	*	**
Cx				-	0,1462
Cb					0,0772

ABCC1	LVCP	4VCP	Cx	Cb	MV
LVCP		-	-	-	0,0642
4VCP			-	-	*
Cx				-	0,1003
Cb					*

ABCC4	LVCP	4VCP	Cx	Cb	MV
LVCP		-	**	**	*
4VCP			**	**	*
Cx				-	-
Cb					-

SLC47A1	LVCP	4VCP	Cx	Cb	MV
LVCP		-	-	*	0,0802
4VCP			-	**	*
Cx				**	*
Cb					-

SLC47A2	LVCP	4VCP	Cx	Cb	MV
LVCP		-	***	*	*
4VCP			***	*	*
Cx				-	-
Cb					-

SLC22A8	LVCP	4VCP	Cx	Cb	MV
LVCP		-	**	**	*
4VCP			**	**	*
Cx				-	-
Cb					

SLCO3A1	LVCP	4VCP	Cx	Cb	MV
LVCP		-	**	0,0606	**
4VCP			**	-	**
Cx				-	-
Cb					0,0606

SLCO2B1	LVCP	4VCP	Cx	Cb	MV
LVCP		-	*	-	*
4VCP			*	-	*
Cx				*	
Cb					*

SLCO1A2	LVCP	4VCP	Cx	Cb	MV
LVCP		-	**	**	-
4VCP			**	**	-
Cx				-	*
Cb					*

SLCO2A1	LVCP	4VCP	Cx	Cb	MV
LVCP		-	*	**	0,0904
4VCP			*	**	-
Cx				-	-
Cb					-

SLC04A1	LVCP	4VCP	Cx	Cb	MV
LVCP		-	0,1043	**	0,0792
4VCP			*	**	*
Cx				0,0697	-
Cb					

TTR	LVCP	4VCP	Cx	Cb
LVCP		-	**	**
4VCP			**	**
Cx				-
Cb				

Supplementary Table 1 : Statistical analysis of qRT-PCR data presented in Figures 1, 2, 4, 6. Stars represents significance. q values close to significance with Q set at 0.05 are also reported. See methods for details. (MV) : Only 2 samples could be analysed.
